# Understanding Bowel Habits and Stool Form Among Indian Adolescents: A Descriptive Study

**DOI:** 10.7759/cureus.79299

**Published:** 2025-02-19

**Authors:** Shashwat Jha, Shalini Verma, Shrish Bhatnagar

**Affiliations:** 1 Pediatrics, Era's Lucknow Medical College & Hospital, Lucknow, IND

**Keywords:** adolescent, bowel habits, bristol stool chart, defecation, stool

## Abstract

Introduction: Bowel habits and stool characteristics reflect adolescents' physical and mental health. Sociodemographics, environment, diet, and genetics influence defecation patterns. However, data on Indian adolescents' normal defecation patterns is limited.

Methods: A study was conducted on 232 adolescents (aged 10-19 years) from two schools after obtaining parental consent and children's assent. Data on bowel habits, stool forms, use of school latrines, socio-dietary habits, and demographic profiles was collected. Statistical analysis was performed using the chi-squared test and unpaired t-test, with p<0.05 considered significant.

Results: Of the 232 participants (mean age: 14.59±2.12 years; 50% male, 50% female), the most common stool frequency was 1-2 times/day (88.8%), followed by thrice a day (4.7%), <3 times/week (3.9%), and >3 times/day (2.6%). Bristol type IV stool was the most prevalent, followed by types III, II, V, I, VI, and VII. Common bowel issues included abdominal pain and bleeding during defecation. Girls reported significantly higher rates of abdominal pain (p=0.009) and incomplete evacuation (p=0.018) compared to boys.

Conclusions: This study provides insights into the normal bowel habits and defecation patterns of urban and semi-urban adolescents in Northern India. The findings can serve as a foundation for larger studies and aid in developing guidelines for managing constipation and bowel irregularities among Indian adolescents.

## Introduction

Stool characteristics, such as color, consistency, and frequency, are important indicators of a person's overall health. The frequency of bowel movements, time of stool passage, and stool mass, color, consistency, texture, and biochemical and microbial composition are scientifically proven stool characteristics useful in determining an individual's health and disease state [1]. Defecation disorders are influenced not only by physical health but also by mental health, which has been shown to impact bowel habits and behavior in growing teenagers [2,3]. The frequency of the pediatric population's bowel movements and defecation habits has a close relationship with their age, sex, and environmental factors such as diet, social background, parental behavior, genetics, and cultural beliefs [4,5].

Despite the significance of bowel habits and stool characteristics for diagnostic purposes, it has remained a less explored subject, especially in adolescent children who, even though they are toilet-independent, remain hesitant about discussing defecation-related issues. Very few studies have examined this important but overlooked topic in the adolescent population [6]. Although pediatric practice addresses diarrhea and constipation more commonly on a day-to-day basis, there is a lack of studies describing normal defecation frequency and stool form in the context of the Indian adolescent population. Therefore, it is important to assess a targeted population's normal bowel movement pattern and have in-depth knowledge of stool frequency so the deviation from the normal is promptly identifiable and treatable [3,7,8].

Hence, this study was planned to evaluate the frequency and form of defecation in adolescent school-going children aged 10-19 in an urban population of Northern India. It aimed to provide a basis for the literature on normal defecation patterns and stool forms in the South Asian adolescent population.

## Materials and methods

This cross-sectional study was conducted in Lucknow, India, over six months, from July to December 2021, after obtaining approval from the institutional ethics committee. The study aimed to assess bowel habits among adolescents and identify associated factors. A total of 232 school- and college-going adolescents aged 10-19 years, from both genders, were included through convenient sampling. The participants were selected from two schools after obtaining proper parental consent and assent from the students. Students who had taken medications known to alter bowel habits, such as stool softeners, antibiotics, and probiotics, in the one month preceding the study were excluded. Those who had undergone any gastrointestinal surgical intervention and students with chronic medical conditions affecting the gastrointestinal system, including celiac disease, inflammatory bowel disease, abdominal tuberculosis, and thyroid disorders, were also excluded to minimize confounding variables.

The sample size was determined using the standard formula for proportion-based calculations, *n=Z^2^×p×q/L^2^*, where *n* is the required sample size,* Z* is the Z-score corresponding to the desired confidence level (1.96 for a 95% confidence interval), *p* is the estimated proportion of students with normal stool form based on previous studies (64.5%), *q* is the complement of *p* (1-p=35.5%), and *L* is the allowable error (4%). After accounting for a potential 10% data loss, the final sample size was determined to be 232 students.

**Figure 1:**
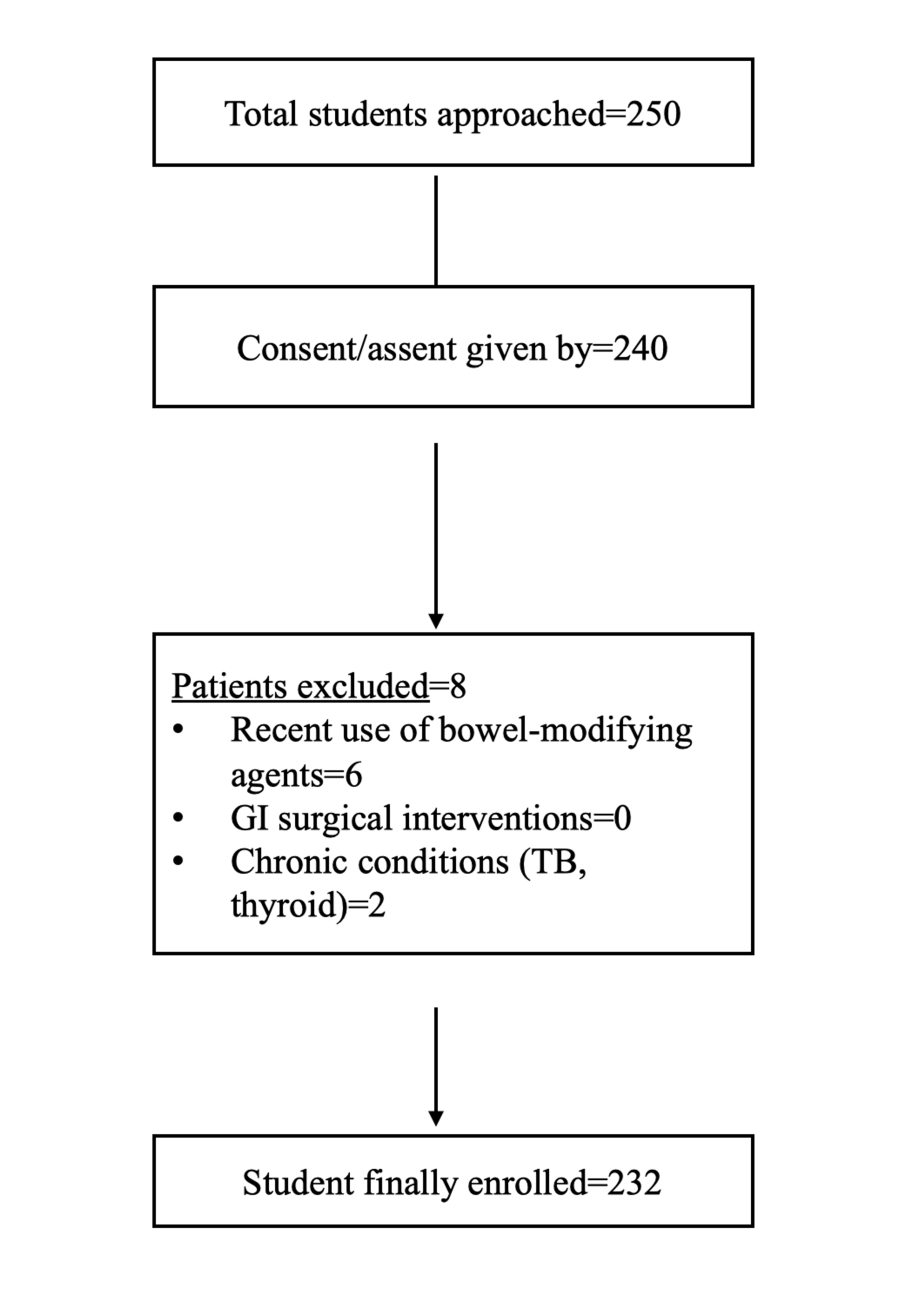
Flowchart of the study (n=232) GI: gastrointestinal; TB: tuberculosis

Before initiating data collection, the study protocol was explained to the principals and teachers of the selected schools to seek their cooperation. On the first visit, a total of 250 students approached; they were given a letter addressed to their parents, detailing the study's objectives and voluntary nature. Parents were required to sign a consent form allowing their children to participate, while assent was also obtained from the students. A total of 240 gave consent/assent to participate, out of which eight were excluded as per the exclusion criteria. A total of 232 students were finally enrolled in the study (n=232) (Figure 1).

To ensure comprehensive understanding, study materials, including the information sheet and consent form, were provided in both Hindi and English. A week later, during the second visit, students who had submitted signed consent forms were given a pre-validated questionnaire, which was pilot-tested on 10 students before commencing the study. The questionnaire was completed under the supervision of the research team. To minimize the potential response bias, the questionnaire was administered under the supervision of the research team, with careful attention to ensuring that participants understood the confidentiality of their responses. The importance of honest and accurate reporting was also emphasized to reduce the possibility of social desirability bias. 

The data was gathered on various parameters, including sociodemographic details, dietary habits, defecation practices, stool frequency over the last four weeks, and stool consistency. Dietary habits included questions on fiber intake, water consumption, and the frequency of junk food consumption. Defecation practices were assessed based on the timing of bowel movements, any associated straining, and the type of toilets used. Stool consistency was evaluated using the Bristol Stool Chart, a validated tool that categorizes stool into seven types, ranging from hard lumps (type I) to entirely liquid stools (type VII). A pictorial representation of the stool types was included in the questionnaire to ensure accurate self-reporting by the participants.

Statistical analysis was performed using IBM SPSS Statistics for Windows, V. 21.0 (IBM Corp., Armonk, NY, USA). Categorical variables were presented as proportions, while continuous variables were expressed as mean±standard deviation (SD). The chi-squared test was used to analyze associations between categorical variables, while the unpaired t-test was applied for comparing continuous variables. A p-value of less than 0.05 was considered statistically significant.

The study was conducted in accordance with ethical guidelines and principles outlined in the Declaration of Helsinki, updated in 2013. Ethical clearance was obtained from the Institutional Ethics Committee of Era's Lucknow Medical College & Hospital under approval number: ELMC&H/RCell,EC/2021/138. Participant anonymity and data confidentiality were strictly maintained throughout the study, and participation was entirely voluntary.

## Results

Of the 232 subjects (age: 14.71±2.38 years, range: 10-19 years), 116 (50%) were male and 116 (50%) were female. Table 1 depicts the sociodemographic profile, dietary habits, and physical activity of the study population.

**Table 1:** Sociodemographic profile, dietary habits, and physical activity duration of the study population

SN	Characteristics	No. of subjects	Percentage (%)
1	Age group (in years)		
	10-12	44	19.0
	13-15	103	44.4
	16-19	85	36.6
	Mean age±SD	14.71±2.38	
2	Gender		
	Male	116	50.0
	Female	116	50.0
3	Level of education		
	Middle (VI-VIII)	67	28.9
	High school (IX-X)	79	34.1
	Intermediate (XI-XII)	86	37.1
4	Diet		
	Vegetarian	103	44.4
	Non-vegetarian	129	55.6
5	Frequency of junk food intake		
	Don't take junk food	40	17.2
	Once a week	105	45.3
	2-3 times a week	72	31.0
	>3 times a week	15	6.5
6	Daily physical activity		
	No physical activity	87	37.5
	<30 minutes of physical activity	62	26.7
	1-2 hours of physical activity	68	29.3
	>2 hours of physical activity	15	6.5

**Figure 2:**
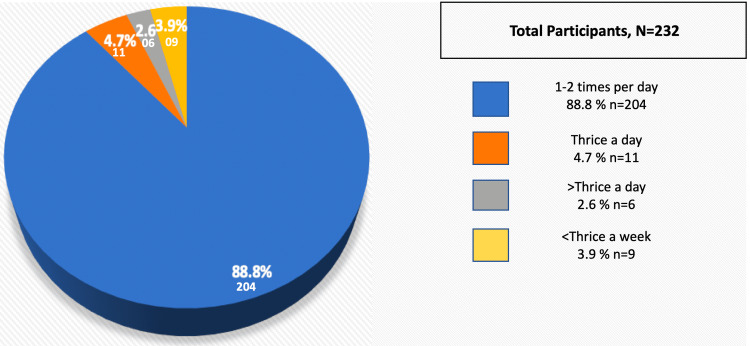
Frequency of defecation among the study population

Most of the study population was from urban areas (89.7%) and used the toilet for defecation (96.6%) in the evening (77.6%), and the commonest stool frequency was 1-2 times/day (88.8%), followed by thrice a day (4.7%), less than three per week (3.9%), and more than thrice a day (2.6%) (Table 2, Figure 2).

**Table 2:** Living area and defecation characteristics of the study population

SN	Characteristics	No. of subjects	Percentage (%)
1	Living area		
	Rural	24	10.3
	Urban	208	89.7
2	Type of defecation practiced		
	Latrine	224	96.6
	Open	8	3.4
3	Number of latrines in-house		
	1	59	25.4
	2	79	34.1
	3	56	24.1
	4+	38	16.4
4	Usual time of defecation		
	Morning	39	16.8
	Evening	180	77.6
	Night	13	5.6

Almost half of the children (117 (50.4%)) refrained from using school toilets, of which most were boys (Table 3). The possible reason could have been poor hygiene and infrastructure and poor privacy.

**Table 3:** Use of school toilets of the study population

SN	Characteristics	No. of subjects	Percentage (%)
1	Restrain to use school toilet		
	Yes	117	50.4
	No	115	49.6
2	Reason of restrain (n=117)		
	Unhygienic	75	64.1
	Uncomfortable	38	32.5
	Lack of facilities (water/locks/no. of toilets)	4	3.4

**Figure 3:**
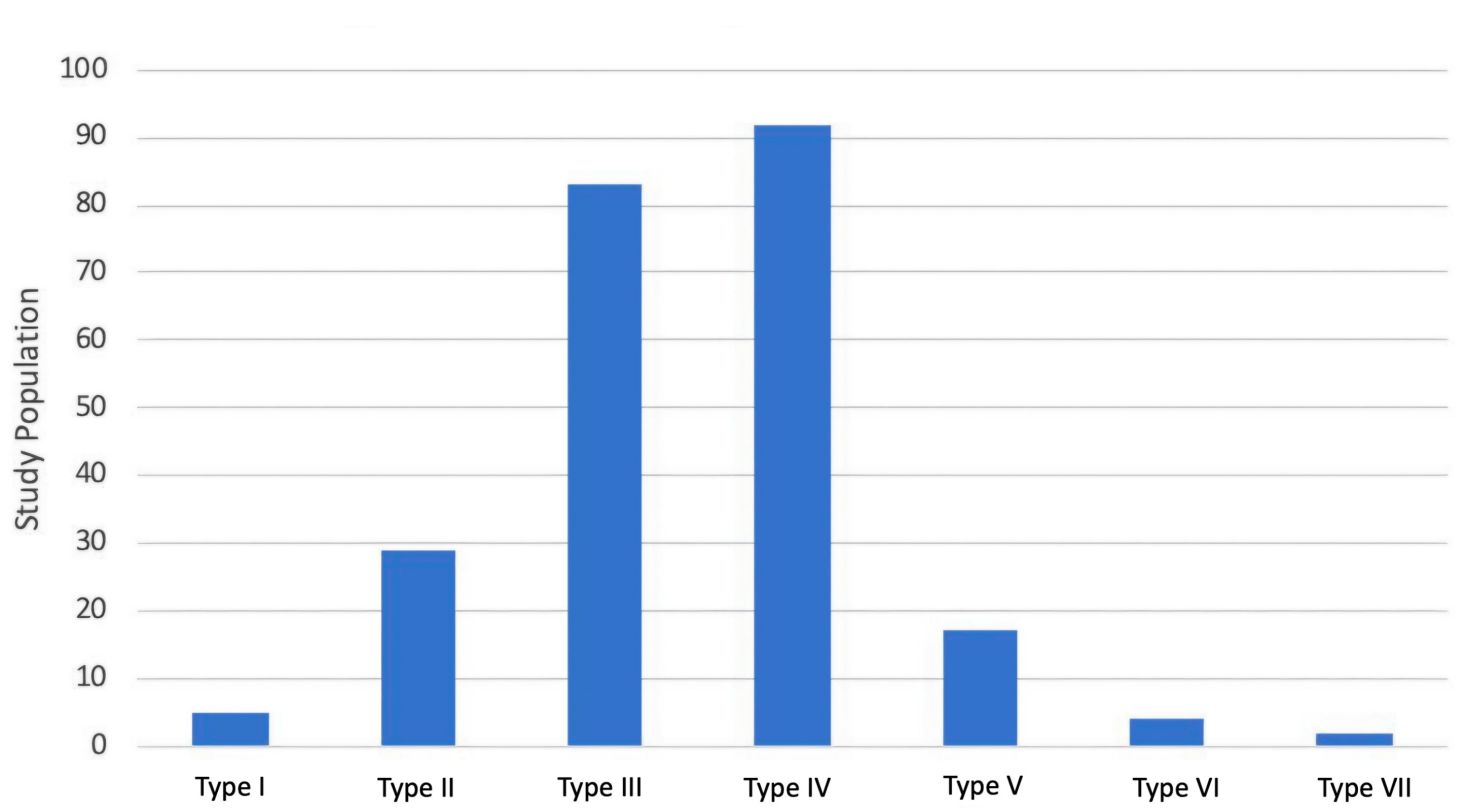
The type of stool forms by the Bristol Stool Chart among the study population

Though a higher proportion of boys found school toilets unhygienic and with a lack of facilities (water, locks, and number of toilets) and a higher proportion of girls restrained from using toilets by the virtue of feeling uncomfortable, the difference was not statistically significant. The most common cause of restraint was unhygienic toilets (64.1%), followed by uncomfortable toilets (32.47%) and lack of facilities (3.41%) (Table 3). Most of the children passed predominantly Bristol type IV stool (92 (39.65%)), followed by type III (83 (28.42%), type II (29 (12.50%)), type V (17 (7.32%)), type I (five (2.15%)), type VI (four (1.72%)), and type VII (two (0.86%)) (Figure 3).

Table 4 shows the comparison of bowel patterns and the associated factors between boys and girls.

**Table 4:** Association of gender with bowel pattern of the study population in the past three months

SN	Characteristics	Total (n=232) (%)	Female (n=116) (%)	Male (n=116) (%)	P-value
1	Abdominal pain	55 (23.7)	36 (31.0)	19 (16.4)	0.009
2	Bleeding during defecation	10 (4.3)	4 (3.4)	6 (5.2)	
3	Painful defecation	53 (22.8)	32 (27.6)	21 (18.1)	
4	Stool size large enough to clog the toilet	16 (7.0)	10 (62.5)	6 (37.5)	
5	Practice of holding toilets	48 (20.6)	26 (22.4)	22 (19.0)	
6	Straining	101 (43.5)	55 (47.4)	46 (39.7)	
7	Passage of mucus	44 (19.0)	25 (21.6)	19 (16.4)	
8	Incomplete evacuation	122 (52.6)	70 (60.3)	52 (44.8)	0.018
9	Consistency				
	Normal	173 (74.6)	84 (72.4)	89 (76.7)	
	Hard	13 (5.6)	6 (5.2)	7 (6.0)	
	Very soft/mushy	8 (3.4)	5 (4.3)	3 (2.6)	
	Changing consistency	38 (16.4)	21 (18.1)	17 (14.7)	
10	Bristol type				
	Type I	5 (2.2)	3 (2.6)	2 (1.7)	
	Type II	29 (12.5)	13 (11.2)	16 (13.8)	
	Type III	83 (35.8)	40 (34.5)	43 (37.1)	
	Type IV	92 (39.7)	50 (43.1)	42 (36.2)	
	Type V	17 (7.3)	8 (6.9)	9 (7.8)	
	Type VI	4 (1.7)	1 (0.9)	3 (2.6)	
	Type VII	2 (0.9)	1 (0.9)	1 (0.9)	
11	Frequency of stool				
	<3/week	9 (3.9)	6 (5.2)	3 (2.6)	
	1-2 times/day	206 (88.8)	103 (88.8)	103 (88.8)	
	3 times/day	11 (4.7)	5 (4.3)	6 (5.2)	
	>3 times/day	6 (2.6)	2 (1.7)	4 (3.4)	

In this study, we assessed problems and unhealthy practices associated with defecation such as abdominal pain, bleeding during defecation, painful defecation, stool large enough to clog the toilet, holding habit, straining, the passage of mucus, and incomplete evacuation reported by 23.7%, 4.3%, 22.8%, 7%, 20.6%, 43.5%, 19%, and 52.6% children. A higher proportion of girls complained of abdominal pain and incomplete evacuation as compared to boys, and this difference was statistically significant with p-values of 0.009 (abdominal pain) and 0.018 (incomplete evacuation). There was no significant difference in the genders for the other parameters.

## Discussion

The present study assessed adolescent children's bowel habits and patterns, evaluating stool frequency and consistency. The target population was Lucknow city, which is a tier-2 city with a diversified sociodemographic profile of inhabitants from multiple religious groups with diversified dietary and lifestyle habits; thus, it gives a true representation of the Indian population. This topic has been studied in different studies of diverse adolescent populations across the globe [9-14]. Most of these studies have shown almost equal representation of boys and girls, similar to ours. The mean age of the study participants in these studies is also comparable to ours (mean age±SD=14.59±2.12). These participants' sociodemographic profiles were studied in detail to understand the problems in the context of their bowel patterns. In our study, most children had a residence in urban areas (89.7%). Similarly, a few other studies have also been done with a focus on sociodemographic context by Devanarayana and Rajindrajith and Akinbami et al. [10,13]. The majority of children were non-vegetarian (55.6%), took junk food 1-3 times per week, and had no or <30 minutes per day physical activity profile (63.2%). Lifestyle and dietary habits are known to have an impact on stool frequency, stool consistency, and other characteristics. These factors have also been studied by Huang et al. and Wu et al. [14,15]. In our study, the majority of children defecated in toilets/latrines (96.6%), defecated in the evening (77.6%), and defecated 1-2 times per day (88.8%). Similarly, a few other studies have reported bowel movements at least once per day in India and globally [10,13,15-17]. In our study, the practice of defecation in toilets/latrines was more common among the urban and semi-urban population, likely influenced by recent government initiatives such as the Pradhan Mantri Swachh Bharat Mission. Epidemiological studies have shown that most defecations occur early in the morning; however, as in our study, a higher proportion of evening defecation can be a lifestyle variation [11]. Maladjusted circadian rhythms in the bowel have been linked to digestive pathologies, including constipation and irritable bowel syndrome [18]. Even though we studied the bowel habits of school-going adolescents in the area of Northern India, we should have looked in detail at the factors that affect bowel habits. In the future, focus groups and in-depth interviews can be used to investigate the variables impacting the bowel habits of this population. Various psychological factors play a key role at this age of physical and emotional growth, which might have an impact on different defecation patterns and practices. We did not investigate this aspect in our study.

In our study, the usage pattern of school toilets and reasons for restraint to use them were assessed too with no significant difference between male and female participants for the reason of restraint (Table 3). Approximately half (50.4%) exercised restraint in using school toilets because they found the toilets unhygienic (64.1%). These findings were in agreement with previous studies done in Vietnam and Kenya [19,20].

According to Bristol stool type, maximum participants (39.7%) had type IV followed by type III (35.8%), type II (12.5%), type V (7.3%), type I (2.2%), type VI (1.7%), and type VII (0.9%) (Figure 3). Sujatha et al. reported the dominance of type III/IV (75.9%) which was comparable to our study (75.5%); however, type I/II was only in 2.9% of children as compared to our study (14.7%), and few other studies have reported similar findings of type III/IV being the dominant type in adolescents [8,21]. Our findings provide valuable insights into this age group; however, further studies in diverse populations are needed to enhance generalizability. In the study by Shiyam et al., type I/II comprised a larger proportion (32.6%), while in our study, type I/II patterns were 14.7% only [17]. They explained that this higher prevalence of type I/II is due to a high proportion of functional constipation (18.7%). We did not make such an assessment, but based on bowel frequency patterns, constipation did not emerge as a major problem.

Table 4 depicts the bowel patterns and associated practices with defecation in our study showing abdominal pain with defecation in 23.7% of participants. Devanarayana and Rajindrajith too reported painful defecation in 10.6% of participants [10]. Similarly, Altamimi and Al-Safadi and Ayonrinde et al. have reported painful defecation as well in 20.6% and 36% of participants, respectively [16,21]. Abdominal pain appears to be one of the most common problems reported in children and should be evaluated in context with bowel irregularities.

In our study, a sizeable proportion of children reported normal stool consistency (74.6%) (Table 4). Contrary to that, few other studies have reported a much higher proportion of normal consistency [10,14]. While it may seem unlikely that nearly half of the participants reported straining and incomplete evacuation despite 74.6% having normal stool consistency, there are several potential explanations. These symptoms may be associated with functional constipation, where individuals experience normal stool but have difficulty fully evacuating due to factors like poor bowel habits, stress, or inadequate pelvic floor relaxation. Additionally, psychosocial factors such as stress, anxiety, or discomfort with using school toilets may contribute to straining and incomplete evacuation, even in the absence of abnormal stool consistency.

Complaints of abdominal pain and incomplete evacuation were statistically significantly higher among girls (p-values of 0.009 and 0.018, respectively), while straining was the most prominent complaint among all (Table 4). However, stool consistency, frequency, and pattern showed no significant association with gender. Devanarayana and Rajindrajith reported a higher prevalence of straining, bulky stools, and withholding posture in girls; similarly, Wu et al. had also observed a higher incidence of constipation in girls as compared to boys [10,15]. The higher prevalence of abdominal pain and incomplete evacuation in girls could be attributed to several physiological, hormonal, psychological, and dietary factors. These factors, either independently or in combination, could explain why girls experience more frequent abdominal pain and incomplete evacuation compared to boys.

Although our study described the bowel habits of school-going adolescents in Northern India, a more detailed assessment of the factors influencing these habits would have provided deeper insights. Future studies could incorporate focus groups and in-depth interviews to explore the variables affecting bowel habits in this population. Psychological factors play a crucial role during this phase of physical and emotional development and may influence defecation patterns and practices. Understanding these underlying influences could help in developing targeted interventions for promoting healthy bowel habits in adolescents.

This study has several strengths, including a large and diverse sample of 232 adolescents from urban areas, which ensures a broad representation of sociodemographic backgrounds and makes the findings applicable to a wider adolescent population in India. The study also provided a comprehensive analysis of dietary habits, physical activity levels, and defecation practices, which were critical in understanding the factors influencing bowel patterns. Furthermore, the use of the Bristol Stool Chart ensured consistent and standardized measurement of stool consistency, allowing for comparison with both Indian and global studies. The real-world relevance of the study is underscored by the urban setting and recent sanitation drives under the Pradhanmantri Sauchalay Yojana. However, the study also has limitations. Being cross-sectional, it cannot establish causality or track changes over time, and the reliance on self-reported data may introduce recall or reporting bias. Moreover, dietary habits, age-related hormonal changes, and psychological factors, such as stress or anxiety, which can significantly impact bowel habits in adolescents, were not explored in detail, and future studies should consider investigating these aspects. Additionally, the study was conducted in Lucknow, a tier-2 city, which may limit the generalizability of the findings to rural or other urban populations. Lastly, while the study assessed stool frequency and consistency, it did not specifically address the prevalence or causes of constipation, a key concern in adolescence, suggesting the need for future research in this area.

## Conclusions

The present study provided valuable insights into stool frequency and consistency among adolescents aged 10-19 years in Northern India, offering a normative reference for clinical practice. Understanding normal bowel patterns in this age group is crucial for identifying deviations that may indicate underlying gastrointestinal disorders. These findings can aid pediatricians and healthcare providers in distinguishing between healthy and unhealthy stool patterns, facilitating early diagnosis and intervention. While this study suggests associations between factors like diet, hydration, and lifestyle and bowel habits, it is important to note that the cross-sectional design does not allow for causal inferences. Further research is recommended to validate these findings and explore potential factors influencing stool patterns. A better understanding of these relationships could help develop targeted interventions to promote gut health in the adolescent population.
